# Short Hairpin RNA (shRNA) Ether à go-go 1 (Eag1) Inhibition of Human Osteosarcoma Angiogenesis via VEGF/PI3K/AKT Signaling

**DOI:** 10.3390/ijms131012573

**Published:** 2012-10-01

**Authors:** Jin Wu, Xinyu Wu, Daixing Zhong, Wenliang Zhai, Zhenqi Ding, Yong Zhou

**Affiliations:** 1 Department of Orthopaedics, The Affiliated Southeast Hospital of Xiamen University, Zhangzhou 363000, China; E-Mails: wuxinyu0102@163.com (J.W.); wlzhai@263.net (W.Z.); zhenqiding175th@yahoo.com.cn (Z.D.); 2 Department of Neurology, The Affiliated Southeast Hospital of Xiamen University, Zhangzhou 363000, China; E-Mail: yuxin0506@163.com; 3 Department of Thoracic Surgery, The Affiliated Tangdu Hospital of Fourth Military Medical University, Xi’an 710038, China; E-Mail: xingxing.1230@hotmail.com; 4 Department of Orthopaedics, The Affiliated Tangdu Hospital of Fourth Military Medical University, Xi’an 710038, China

**Keywords:** Ether à go-go1 (Eag1), vascular endothelial growth factor (VEGF), proliferation, angiogenesis, short hairpin RNA(shRNA), osteosarcoma

## Abstract

Ether à go-go 1 (Eag1) channel is overexpressed in a variety of cancers but the therapeutic potential of Eag1 in osteosarcoma remains elusive. In this study, we constructed an Ad5-Eag1-shRNA vector and evaluated its efficiency for Eag1 knockdown and its effects on osteosarcoma. Our results showed that Ad5-Eag1-shRNA had high interference efficiency of Eag1 expression and suppressed osteosarcoma growth both *in vitro* and *in vivo.* To explore the molecular mechanism underlying tumor growth inhibition induced by Eag1 silencing, the intratumoral microvessel density (MVD) was assessed by CD31 staining and the expression of vascular endothelial growth factor (VEGF) was detected by Western blot analysis. We found that Eag1 silencing led to decreased angiogenesis and VEGF expression in the xenograft model of osteosarcoma. Finally, we detected a time-dependent decrease in VEGF expression and considerably reduced phosphoinositide 3-kinase (PI3K) and protein kinase B (AKT) activation in osteosarcoma cells treated by Eag1 shRNA. Taken together, our results suggest that Eag1 silencing inhibits tumor growth and angiogenesis in osteosarcoma via the down regulation of VEGF/PI3K/AKT signaling.

## 1. Introduction

Voltage-gated potassium channels (Kv) perform many vital functions in both electrically excitable and nonexcitable cells. In recent years, the importance of Kv channels in tumor biology has been an area of intense investigation [1]. The Ether à go-go 1 (Eag1 (Kv10.1, KCNH1)) channel is a member of Kv channels that has been implicated in tumor growth, progression and metastasis [2,3]. However, the molecular mechanisms responsible for the oncogenic potential of Eag1 remain elusive [4,5].

Angiogenesis not only plays an important role in physiological processes, but also contributes to the pathology of a number of diseases, especially cancer [6]. Recently, the concept of the “angiogenic switch” was proposed to explain how a tumor develops and metastasizes [7]. Eag1 appears to induce tumor angiogenesis by the release of hypoxia inducible factor-1 (HIF-1) and vascular endothelial growth factor (VEGF) upon hypoxia [8].

Although Eag1 is overexpressed in a variety of cancers, the therapeutic potential of Eag1 in osteosarcoma remains elusive. In this study, we designed a short hairpin RNA (shRNA) targeting Eag1 and evaluated its effects on osteosarcoma growth and angiogenesis. Our results showed that Eag1 silencing significantly inhibited osteosarcoma growth both *in vivo* and *in vitro*. Furthermore, we demonstrated that Eag1 shRNA inhibited osteosarcoma angiogenesis and this is associated with the downregulation of the VEGF/PI3K/AKT signaling.

## 2. Results

### 2.1. Knockdown Efficiency of Ad5-Eag1-shRNA

**Figure 1:**
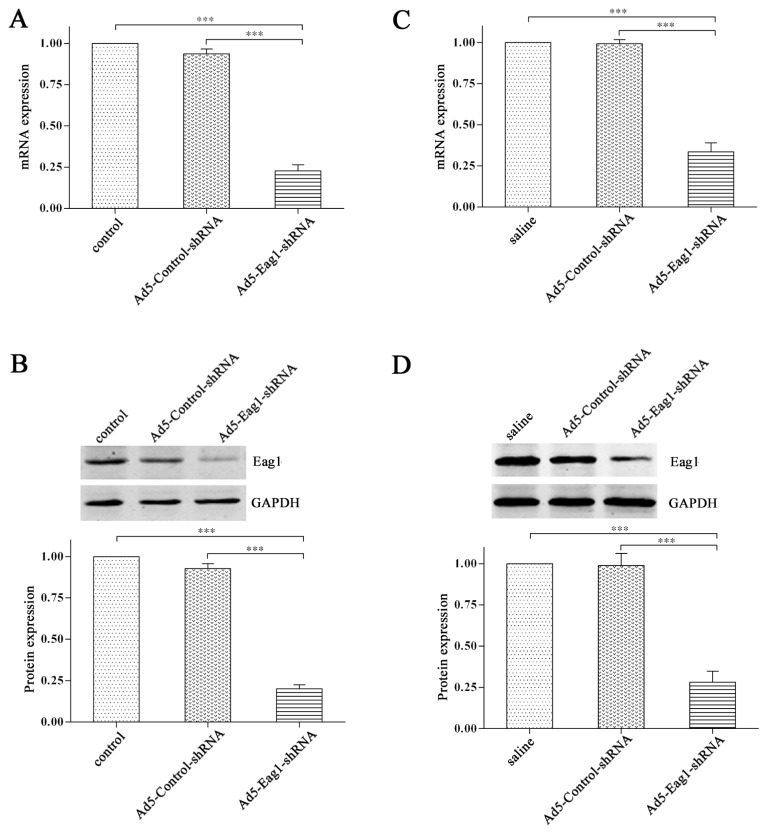
High interference efficiency of Ad5-Eag1-shRNA. (**A**) *In vitro* experiment showed that the expression of Eag1 mRNA in the Ad5-Eag1-shRNA group was significantly lower than that in the control group or Ad5-Control-shRNA group. (**B**) Grey-value analysis of western bolt was completed using GAPDH as the internal reference, and the results were expressed as mean ± SD (*n* = 3). (**C**,**D**) Similar results were obtained from *in vivo* experiment. *** *p* < 0.001.

We constructed adenoviral shRNA vectors Ad5-Eag1-shRNA to inhibit Eag1 expression. The knockdown efficiency was tested by real-time PCR and Western blot analysis. The results showed that the expression levels of Eag1 mRNA (Figure 1A) and protein (Figure 1B) were significantly decreased in Ad5-Eag1-shRNA group (cells infected with Ad5-Eag1-shRNA), compared to control group (cells untreated) or Ad5-Control-shRNA group (cells infected with Ad5-Control-shRNA). The knockdown efficiency of Ad5-Eag1-shRNA was then examined *in vivo*. As shown in Figure 1C,D, Eag1 expression was suppressed obviously in Ad5-Eag1-shRNA injected animals compared to saline or Ad5-Control-shRNA injected animals. Collectively, the results demonstrated the high knockdown efficiency of Ad5-Eag1-shRNA both *in vitro* and *in vivo*.

### 2.2. Eag1 Silencing Inhibits Osteosarcoma Growth *in Vivo*

**Figure 2:**
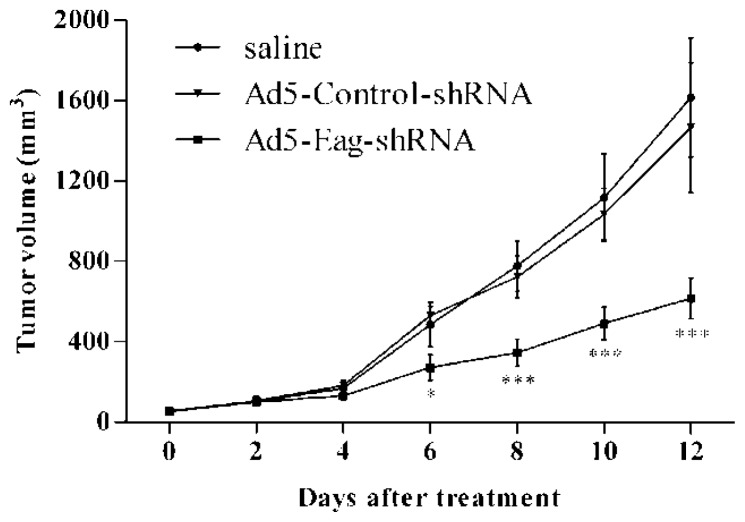
Eag1 silencing inhibits the growth of a MG-63 derived tumor in nude mice. Intra-tumor injection of Ad5-Eag1-shRNA significantly reduced the size of MG-63 derived tumor implanted subcutaneously in nude mice during the 12-day follow-up period as compared with the saline control and the Ad5-Control-shRNA. * *p* < 0.05, *** *p* < 0.001, compared with either the saline control or the Ad5-Control-shRNA (*n* = 6).

To address the potential effects of Eag1 silencing on tumor growth *in vivo*, we made a xenograft model of osteosarcoma on nude mice and treated the xenografts by intra-tumor injection of Ad5-Eag1-shRNA, Ad5-Control-shRNA and saline, respectively. The results showed that the tumor volume was significantly smaller in Ad5-Eag1-shRNA injected animals compared to Ad5-Control-shRNA or saline injected animals (Figure 2). The data demonstrate that Eag1 silencing inhibits osteosarcoma growth *in vivo*.

### 2.3. Eag1 Silencing Inhibits Angiogenesis of Xenografted Osteosarcoma

**Figure 3:**
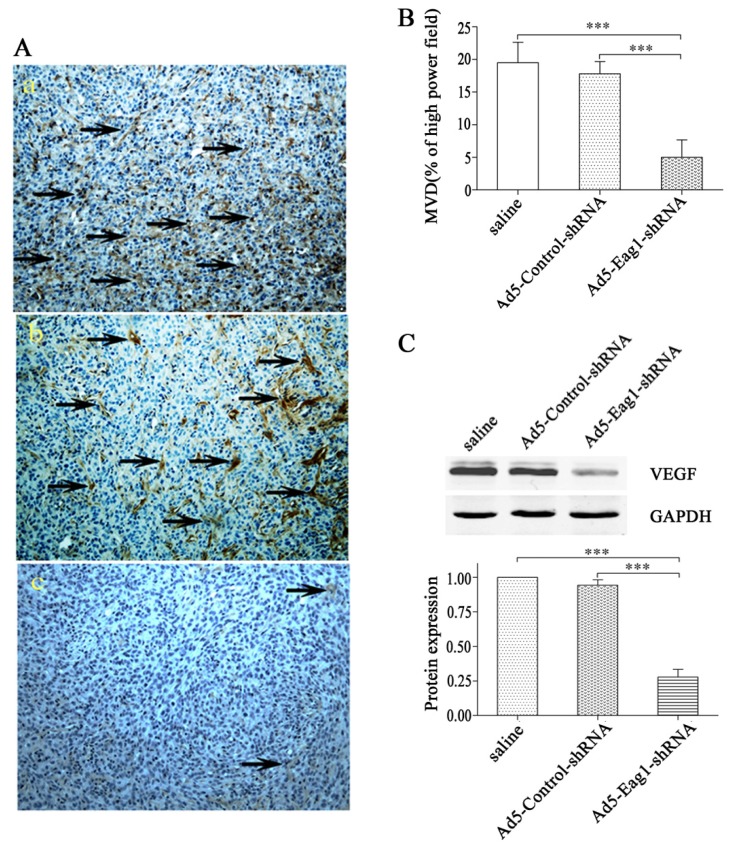
Eag1 silencing reduces intratumoral blood vessels in osteosarcoma-bearing mice. (**A**) 12 days after nude mice receiving injections of saline (**a**), Ad5-Control-shRNA (**b**) or Ad5-Eag1-shRNA (**c**), respectively, the mice were sacrificed. Blood vessels in the microphoto-graphs were marked by a yellow brownish color (anti CD31, black arrow). (**B**) Quantification of the intratumoral MVD by measurement of CD31 in 10 high power fields. *** *p* < 0.001. (**C**) VEGF expression in tumor tissues of nude mice was detected by Western blot analysis. Grey-value analysis was completed using GAPDH as the internal reference, and the results were expressed as mean ± SD (*n* = 3). *** *p* < 0.001.

Next we investigated the potential effects of Eag1 silencing on osteosarcoma angiogenesis. Using xenografted osteosarcoma as the model, we found that Ad5-Eag1-shRNA reduced intratumoral MVD counts based on CD31 immunohistochemistry (5.0 ± 2.7 tumor vessels per high power field, *n* = 10) compared to Ad5-Control-shRNA or saline (17.8 ± 1.9 and 19.5 ± 3.1 tumor vessels per high power field, respectively, *n* = 10, *p* < 0.001, Figure 3A). Quantification of MVD by measurement of CD31 in 10 high power fields from at least two tumors of each type confirmed decreased vascularization in Ad5-Eag1-shRNA group (Figure 3B). Since VEGF is one of the most potent angiogenic factors, we detected the expression of VEGF in tumor tissues of nude mice. Western blot analysis showed that VEGF expression was suppressed in Ad5-Eag1-shRNA group compared with Ad5-Control-shRNA group or saline group (Figure 3C), indicating that the anti-tumor effects of Eag1 silencing on osteosarcoma are related to decreased angiogenesis and reduced VEGF expression.

### 2.4. Eag1 Silencing Reduces the Proliferation of Osteosarcoma Cells

**Figure 4:**
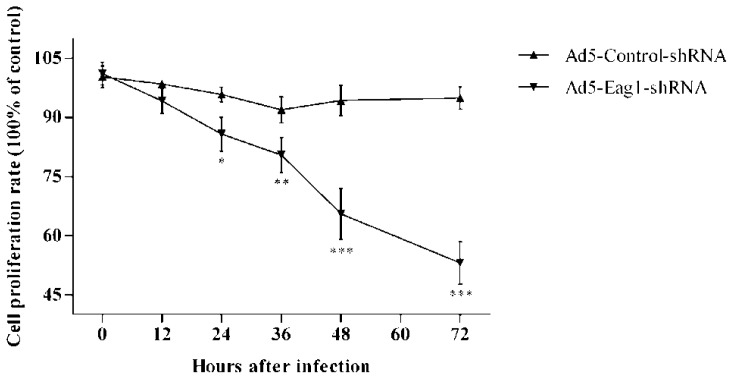
Eag1 silencing inhibits the proliferation of MG-63 cells. CCK-8 assay showing that the proliferation of MG-63 cells was significantly reduced after infection with Ad5-Eag1-shRNA. Data were normalized using the values obtained for untreated cells (negative control, NC) and presented as mean ± SD (*n* = 6). * *p* < 0.05, ** *p* < 0.01, *** *p* < 0.001.

To confirm that Eag1 has similar anti-tumor effects on osteosarcoma cells *in vitro*, we treated MG-63 cells with Ad5-Eag1-shRNA. The results showed that MG-63 cell proliferation was inhibited significantly in Ad5-Eag1-shRNA group compared to Ad5-Control-shRNA group (Figure 4). These *in vitro* data complement our *in vivo* results and confirm the oncogenic role of Eag1 in osteosarcoma.

### 2.5. Eag1 Silencing Inhibits VEGF/PI3K/AKT Signaling in Osteosarcoma Cells

**Figure 5:**
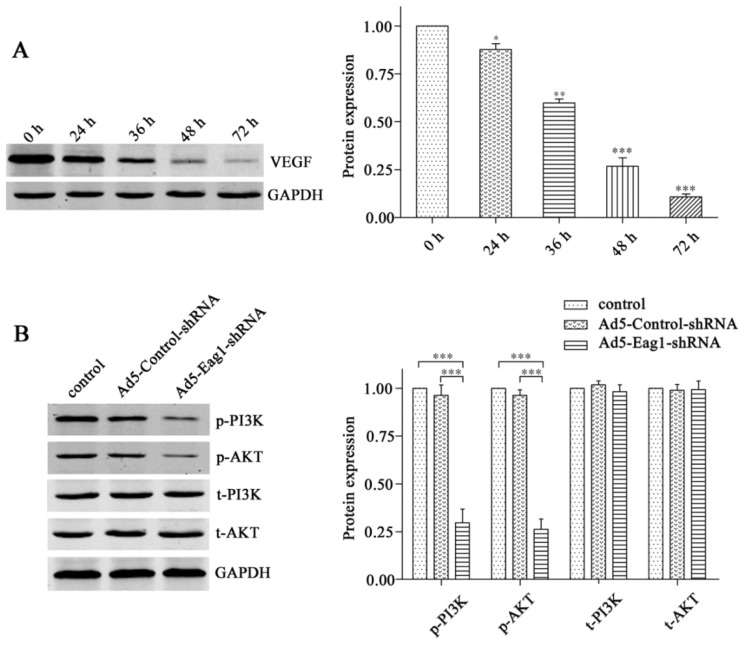
Eag1 silencing inhibits the expression of VEGF and the activation of PI3K and AKT in MG-63 cells. (**A**) Western blot analysis of VEGF levels at different time points following treatment with Ad5-Eag1-shRNA in MG-63 cells. * *p* < 0.05, ** *p* < 0.01, *** *p* < 0.01. *n* = 3. (**B**) Western blot analysis of p-PI3K, p-AKT, t-PI3K and t-AKT levels in control, Ad5-Control-shRNA and Ad5-Eag1-shRNA group. Grey-value analysis was completed as described above. *** *p* < 0.001. *n* = 3.

PI3K/AKT signaling is one of the major downstream intracellular pathways that mediate the biological effects of VEGF [9]. In addition, a substantial number of studies support the important role of VEGF/PI3K/AKT signaling in tumor progression [10,11]. To investigate whether Eag1 silencing was able to inhibit intracellular signal transduction of VEGF, we first detected the expression of VEGF in MG-63 cells at different time points after Ad5-Eag1shRNA treatment. As shown in Figure 5A, a time-dependent decrease in VEGF expression was observed. Then the total PI3K (t-PI3K) and AKT (t-AKT) and the activation of PI3K (phospho-PI3K, p-PI3K) and AKT (phospho-AKT, p-AKT) was detected by Western blot analysis in MG-63 cells. The results showed that Ad5-Eag1-shRNA treatment did not affect the expression of t-PI3K or t-AKT, but considerably reduced PI3K and AKT activation in MG-63 cells, compared to Ad5-Control-shRNA or normal control. (Figure 5B).

## 3. Discussion

Osteosarcoma is the most common primary bone tumor in childhood and adolescence [12,13]. With the improvements in osteosarcoma therapy, 5-year survival rates for patients without metastatic disease are 65% [14]. However, approximately 40%–50% of patients will develop metastases, especially pulmonary metastases, and few of them will be cured [15]. Therefore, it is urgent to develop new treatment strategies for osteosarcoma in the clinic.

Tumor metastasis is a multi-step process in which many factors that can potentially influence tumor dissemination [16,17]. Angiogenesis or neovasculization is required to sustain primary tumor growth as well as metastasis [18]. VEGF is a potent angiogenic factor that contributes to the generation and preservation of tumor vasculature [19,20]. VEGF elicits a pronounced angiogenic response that acts via 3 related receptor tyrosine kinases (RTKs): VEGFR-1, VEGFR-2 and VEGFR-3 [21–23]. Upon VEGF interaction with the extracellular domain of the receptor, dimerization and autophosphorylation of the intracellular receptor tyrosine kinases occurs and a variety of downstream signaling pathways are activated among which PI3K/AKT signaling plays important role in tumor progression and metastasis [10,11]. However, it is unclear whether this signaling pathway plays similar role in osteosarcoma.

In the present study, we first examined the knockdown efficiency by Ad5-Eag1-shRNA and found that it silenced Eag1 expression *in vitro* and *in vivo*. We then analyzed the anti-tumor effects of the Eag1 silencing on osteosarcoma *in vitro* and *in vivo.* The results showed that Eag1 silencing could efficiently inhibit osteosarcoma growth. To address the detail mechanisms of tumor inhibition induced by Eag1 silencing, we focused on angiogenesis. We found that Eag1 silencing not only suppressed angiogenesis in osteosarcoma but also reduced the expression of VEGF. Next, we evaluated the effects of Eag1 silencing on VEGF signaling pathways. The data showed considerably reduced expression levels of VEGF, p-PI3K and p-AKT, but not t-PI3K or t-AKT in osteosarcoma cells after treatment with Ad5-Eag1-shRNA.

Collectively, this study provides the first lines of evidence that Eag1 silencing inhibits tumor growth and angiogenesis in osteosarcoma and these are associated with the downregulation of VEGF/PI3K/AKT signaling. These results provide the basis for the development of therapeutic strategy targeting angiogeneic property of osteosarcoma and will help improve the survival rate of osteosarcoma patients.

## 4. Experimental Section

### 4.1. Cell Culture

Human osteosarcoma cell line MG-63 and human embryonic kidney cell line 293 (HEK293) were purchased from the American Type Culture Collection. MG-63 and HEK293 cells were cultured in RPMI-1640 medium supplemented with 10% fetal bovine serum (FBS) (Gibco, Rockville, MD, USA), 100 U/mL penicillin, and 100 μg/mL streptomycin in a humidified atmosphere of 5% CO_2_ in the air at 37 °C. All cells were subcultured every 3–4 days.

### 4.2. Preparation of Adenoviral shRNA Vectors

The oligonucleotides targeting human Eag1 was designed and selected as the template: AGC CAT CTT GGT CCC TTA TAA, which shared no homology with other coding sequences in human by BLAST analysis. A ring sequence of 9 base pairs (TTC AAG ACG) existed between the sense and antisense strands. The shRNA was synthesized by Sangon Biotech (Shanghai, China). Plasmid pGeneSil-1 was purchased from GeneSil Biotechnology (Wuhan, China). The shRNA-expressing cassette was subcloned into the pAdTrack vector between the HindIII and the XbaI sites [24]. The recombinant plasmid was linearized by digestion with restriction endonuclease and subsequently co-transformed into *E. coli* BJ5183 cells with an adenoviral backbone plasmid, pAdEasy-1. Recombinant plasmids were selected for kanamycin resistance, and transduced into the HEK293 cells. A recombinant adenovirus expressing shRNA against Eag1 (Ad5-Eag1-shRNA) was generated. Ad5-Control-shRNA, with the insertion of random sequences CTA GGT GTT CTA GTC TGG ACT was generated as control. All viruses were propagated and purified on a CsCl gradient using standard methods. The viruses were titered for viral particles using standard methods based on spectrophotometry at 260 nm. Functional titer (plaque forming units) was determined with plaque assay on HEK293 cells.

### 4.3. Tumor Model

Thymys-null BALB/c nude mice (female, 6–8 weeks old) were obtained from the Animal Center of Chinese Academy of Medical Sciences. All animal procedures were performed according to approved protocols and in accordance with recommendations for the proper use and care of laboratory animals. Osteosarcoma xenografts were established in nude mice as described previously [25]. A total of 1 × 10^6^ MG-63 cells in 150 μL Phosphate-buffered Saline (PBS) were subcutaneously injected into the right hind leg. One week later, the tumors had grown to visible size. The osteosarcoma-bearing mice were randomly divided into 3 groups (6 in each group). Group 1 received intra-tumor injections with Ad5-Eag1-shRNA at 10 MOI (multiplicity of infection, calculated as PFU/cell numbers) every 2 days (6 injections totally). Group 2 received intra-tumor injections of Ad5-Control-shRNA (10 MOI) every 2 days. Group 3 received normal saline injection as controls. Tumor volume (cm^3^) was determined based on the following formula: ab^2^/2 where *a* was the length and *b* was the width of the tumor [25].

### 4.4. Histological Sections and Immunohistochemistry

Four-micrometer sections of 10% formalin-fixed, paraffin-embedded tumor tissues were deparaffinized in xylene and rehydrated in ethanol. Afterwards, antigen retrieval was performed by heating the slides for 30 min in a steamer in 10 mM citrate buffer solution pH 6.0. Then slides were incubated overnight in a humidified chamber at 4 °C with anti CD31 antibody (Abcam, Cambridge, MA). Diaminobenzamidine (DAB) (Sangon, Shanghai, China) was used to visualize the tissue slide and the sections were counterstained with haematoxylin. The blinded operator performed the quantification of angiogenesis using ImageJ software on 10 high power fields (200×) from each sample acquired.

### 4.5. Adenovirus Infection

MG-63 cells (1 × 10^5^) in serum-free RPMI-1640 were infected with Ad5-Eag1-shRNA or Ad5-Control-shRNA at 5 MOI in a humidified atmosphere of 5% CO_2_ at 37 °C. Virus-containing medium was removed 8 h later and replaced with fresh RPMI-1640 medium containing 10% FBS. Cells were incubated for another 48 h.

### 4.6. Cell Proliferation Assay

The cell proliferation was analyzed by using Cell Counting Assay Kit-8 (CCK-8) (Dojindo Molecular Technologies, Gaithersburg, MD) according to the manufacturer’s protocol. In brief, 5 × 10^3^ cells were starved in serum-free medium for 12 h and then the cells were transduced. After 0, 12, 24, 36, 48 and 72 h, the cells were harvested. Ten microliters of Cell Counting Assay Kit-8 solution was added to each well, the cells were incubated for another 1 h, and the absorbance (A) at 450 nm was measured by using the automatic multiwell spectrophotometer (Bio-Rad, Richmond, CA). Experiments were performed at least three times with representative data presented.

### 4.7. Real-Time PCR

The total RNA was isolated from the cultured cells or tumor tissues of nude mice by Trizol reagent (Invitrogen, Rockville, MD, USA). Real-time PCR was carried out using LightCycler^®^ 480 Probes Master kit (Roche Diagnostics, Mannheim, Germany), according to the following protocol: DNA denaturation at 95 °C for 10 min, followed by 45 amplification cycles consisting of 10 s at 95 °C, 30 s at 60 °C, and 1 s at 72 °C. Primers sequences were designed as previously described [26]. GAPDH was used as an internal control.

### 4.8. Western Blot Analysis

5~6 × 10^7^ cells or tumor tissues of nude mice were collected and lysed in ice-cold lysis buffer containing 50 mmol/L Tris-Cl (pH 7.5), 150 mmol/L NaCl, 0.2 mmol/L EDTA, 1 mmol/L PMSF and 1% Nonidet-P40 for 30 min. The lysates were centrifuged at 13,200 rpm for 10 min at 4 °C and the supernatants were collected. 50 μg protein were resolved by a 12% SDS-PAGE and blotted on nitrocellulose membranes (Bio-Rad). Membranes were blocked with 10% nonfat milk powder at room temperature for 1 h, and then incubated with antibodies to Eag1 (Polyclonal antibody, Alomone laboratories, Jerusalem, Israel), VEGF, p-PI3K, p-AKT (Catalogue No: sc-7985-R, Santa Cruz Biotechnology, CA, USA), t-PI3K (Santacruz Biotechnology), t-AKT (Cell Signaling Technology^®^, Danvers, MA) and GAPDH (Santacruz Biotechnology) overnight, followed by incubation with horseradish peroxidase-conjugated goat anti-rabbit or anti-mouse secondary antibody (Santacruz Biotechnology). Then the membranes were developed with a chemiluminescent detection kit (Zhongshan Biotechnology, Beijing, China) and exposed to X-ray films. Experiments were performed at least three times with the representative data presented.

### 4.9. Statistical Analysis

All data were presented as mean ± standard error (SD). Statistical significance was determined using *t*-test or analysis of variance (ANOVA) using the SPSS18.0 program. *p* < 0.05 was considered as statistically significant difference.
